# Psychosomatic problems in dentistry

**DOI:** 10.1186/s13030-016-0068-2

**Published:** 2016-04-30

**Authors:** Akira Toyofuku

**Affiliations:** Psychosomatic Dentistry, Graduate School Tokyo Medical and Dental University (TMDU), 1-5-45 Yushima, Bunkyo-ku, Tokyo, 113-8549 Japan

**Keywords:** Psychosomatic dentistry, Medically unexplained oral symptoms, Burning mouth syndrome, Atypical odontalgia, Phantom bite syndrome, Oral cenesthopathy, Halitophobia

## Abstract

Many dental patients complain of oral symptoms after dental treatment, such as chronic pain or occlusal discomfort, for which the cause remains undetermined. These symptoms are often thought to be mental or emotional in origin, and patients are considered to have an “oral psychosomatic disorder”. Representative medically unexplained oral symptoms/syndromes (MUOS) include burning mouth syndrome, atypical odontalgia, phantom bite syndrome, oral cenesthopathy, or halitophobia. With an increasing prevalence of these MUOS, dentists are being asked to develop new approaches to dental treatment, which include taking care of not only the patient’s teeth but also the patient’s suffering. Progress in the understanding of mind-body interactions will lead to investigations on the pathophysiology of MUOS and the development of new therapeutic approaches.

## Background

Many dental patients complain of oral symptoms after dental treatment, such as chronic pain or occlusal discomfort, for which the cause remains undetermined [1]. Such symptoms are often thought to be mental or emotional in origin, and patients are considered to have an “oral psychosomatic disorder” [2]. The clinical need for a psychosomatic approach has been acknowledged for over 100 years by Japanese dentists. However, the mechanical and surgical aspects of dentistry have impeded the development of such an approach. Dentists have inherent difficulties with oral symptoms of undetermined etiology, including toothaches of unknown origin. We tend to look for the most likely cause and repeat ineffective dental treatments that fail to relieve the patient’s symptoms. We finally conclude that the pain “must be psychogenic” and try to avoid patients with psychogenic pain. The Japanese health care insurance system, which only covers conventional dental problems, has contributed to the tendency of dentists to avoid patients with unexplained dental pain, in part because of economic reasons.

Some Japanese dentists have decided to specialize in psychosomatic dentistry. Fortunately, because of increasing knowledge about brain functions, there has been some progress in understanding the mind-body interactions. In 2015, the theme of the 30th anniversary meeting of the Japanese Society of Psychosomatic Dentistry was “From Brain to Dentistry”.

In this article, we introduce psychosomatic problems in dentistry by describing representative “oral psychosomatic disorders” and discuss possible future developments in this field.

### Medically unexplained oral symptoms (MUOS)

Patients who complain of physical symptoms without identifiable etiologies are common in clinical medical practice [3]. Such symptoms are known as medically unexplained symptoms (MUS) and are problematic for many physicians.

In oral medicine, many dentists encounter very similar problems. The most typical symptom is chronic oral pain with “nothing the matter” [4], which is a manifestation of burning mouth syndrome (BMS) [5] and atypical odontalgia (AO) [6]. Complaints about dental occlusion are peculiar to dentistry. Dry mouth or disturbances in taste and salivation may be common problems that other specialists such as otolaryngologists see.

**Fig. 1:**
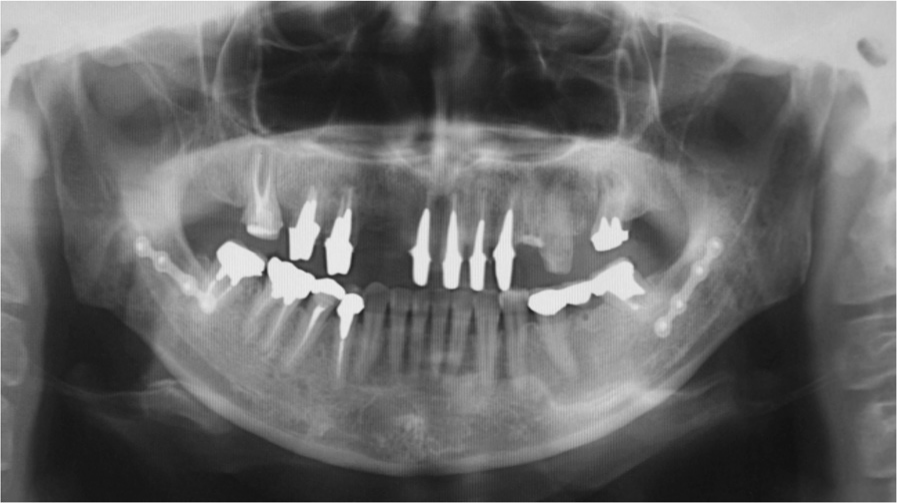
Panoramic radiograph of a patient with phantom bite syndrome. Several dentists had treated a 40-year-old woman for her malocclusion over many years, performing not only prosthodontic procedures but also orthognathic surgery on her mandible. No one was able to treat her to her satisfaction

Dentists tend to overtreat these patients, and excessive or unnecessary dental procedures may worsen them (Fig. 1). Orthopedic surgeons who treat chronic low back pain may encounter similar situations. In the absence of an effective management strategy, the patient’s atypical illness, along with help-seeking behaviors and worries about an unrecognized illness, persist, while frustration and tensions escalate between the dentist and patient [4].

The problems of these patients have been called “oral psychosomatic disorders”; but because of the implication that the problem is “psychogenic”, patients are reluctant to accept the diagnosis. Therefore, the use of another term, “medically unexplained oral symptoms” (MUOS) is preferable. We have reported that the estimated prevalence of MUOS among dental patients ranges from 5 to 10 % or more [2]. Representative MUOS are shown in Table 1.

**Table 1:** Medically Unexplained Oral symptoms/syndromes (MUOS)

1.	Burning Mouth Syndrome (BMS) ^a^
2.	Atypical Odontalgia (AO)
3.	Oral Cenesthopathy (Oral Dysesthesia)
4.	Halitophobia (Olfactory reference syndrome)
5.	Occlusal dyscomfort (Phantom Bite Syndrome)
6.	Odontophobia (Dental Phobia)

^a^BMS includes dry mouth and dysgeusia

### Chronic oral pain

The impact of chronic oral pain on quality of life should not be ignored [7, 8]. BMS and AO are both chronic pain disorders that occur in the absence of any organic cause, and they are often regarded as psychogenic conditions. Although many studies have been performed on the relationship between oral pain and psychological factors, the nature of the relationship remains unclear [9–12]. Few patients with chronic oral pain are treated by psychiatrists [13].

BMS is characterized by a burning sensation involving the tongue or other oral sites, usually in the absence of clinical and laboratory findings [5]. Delays in the diagnosis, referral, and appropriate management of BMS patients are frequent [14]. Patients with BMS are often finally told that “nothing is wrong” [4], even though they have severe pain and have not received any effective treatment. They become frustrated, very anxious, and worried about accruing debt for serious diseases such as oral cancer.

BMS is a syndrome that is manifested by not only pain, but also by many other intra and/or extra oral discomforts [15, 16], including sore mouth, sensation of dry mouth without hyposalivation, and loss of taste or changes in taste, such as a bitter or metallic tastes.

Medications that act in the brain, such as benzodiazepines, tricyclic antidepressants (e.g., amitriptyline), and anticonvulsants (clonazepam) are known to be effective for patients with BMS [17]. SNRIs [18] and SSRIs [19] are also reported to be effective for BMS. Burning mouth pain usually responds to lower dosages in the recommended ranges [5]. Some patients also appear to respond better to low-dose combinations of these medications [17]. But because of the heterogeneity of the pharmacological effects of these drugs, patients have varied responses and a variety of side effects, which hinder dentists in providing adequate prescriptions.

Whether the peripheral or central nervous system underlies BMS remains controversial [20]. Evidence from various clinical and brain imaging studies [21, 22] should enable the subdivision of patients into treatment subgroups based on underlying neurological involvement.

Compared to BMS, AO is not as commonly seen in other medical settings, and it has received substantial attention from dentists in recent years [5, 23]. The International Association for the Study of Pain defines AO as severe throbbing pain in a tooth without major pathology. Ineffective treatment of this type of chronic dental pain often is considered to be treatment failure, which results in repeated, ineffective dental treatments to relieve the pain, such as dental filling, root canal, or even extraction. The resulting iatrogenic changes to the treated tooth leads to difficulty in performing further diagnostic evaluations.

The lack of knowledge about the pathophysiologic mechanisms of these pain conditions is a major reason for problems in their diagnosis and management [20]. The underlying pathophysiologic mechanism seems to be neural dysfunction triggered by some type of dental or oral manipulation that involves the peripheral sensory nerves or the region of the brain that processes the sensation of oral pain.

Amitriptyline is one of the more commonly prescribed tricyclic medications for AO [23]. Because of the many side effects and varied responses to this drug, very few dentists prescribe it. Although psychotherapy may be needed in some cases, in many cases it alone does not result in satisfactory improvement. Dental education should add additional training on the pharmacotherapies for AO.

Although BMS and AO both manifest as chronic oral pain, they differ in other clinical characteristics. For example, the mean age of patients with AO is significantly lower than the mean age of BMS patients, which indicates possible differences in biologic background [11]. However, for the pathophysiologic mechanisms underlying the chronic oral chronic pain of both BMS and AO, ongoing basic investigations in dentistry are focused on the peripheral (trigeminal) nerves [24, 25].

### Occlusal discomfort

**Fig. 2:**
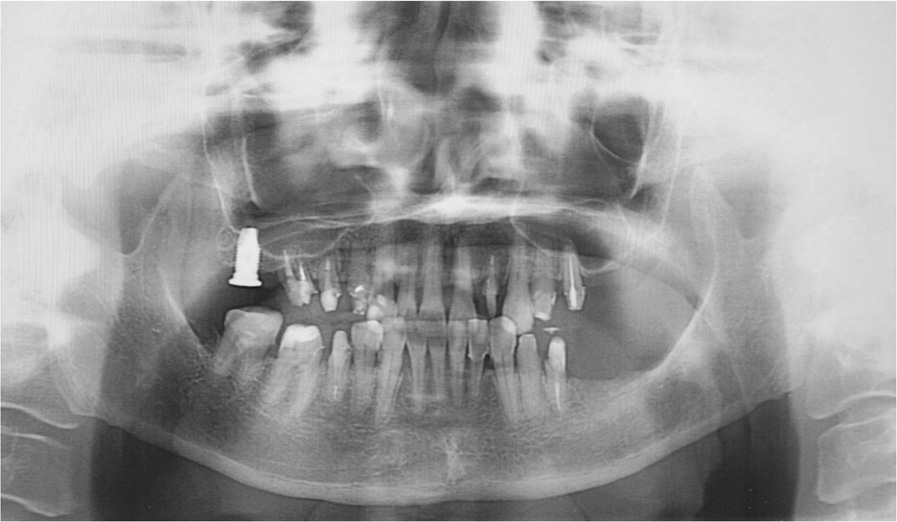
Panoramic radiograph of a patient with phantom bite syndrome. This 70-year-old woman had been complaining of occlusal dyscomfort and had visited various dentists for “bite correction”, but had never finished the treatments

Occlusal discomfort is a problem unique to dentistry, and some patients irritate their dentists because of their unreasonable complaints, demands, and incomprehensible claims concerning dental treatment. Phantom bite syndrome (PBS) is characterized by a persistent, uncomfortable sensation of occlusion without any evidence of occlusal discrepancy [26–28]. PBS is also called “occlusal discomfort” or “occlusal dysesthesia” [29, 30]. Patients complain that their occlusion is “wrong”, “somewhat high/low”, or “the bite is off.” They visit multiple dentists seeking “bite correction” because of their strong belief in dental treatment, regardless of the risk of exacerbating their symptoms (Fig. 2) [31]. Various MUS, including headache, dizziness, shoulder stiffness, low back pain, and fatigue, often accompany PBS and worsen after even minor dental adjustments, which the patients have requested [32].

Because of the ineffectiveness of repeated occlusal adjustments, PBS has been regarded to be a psychiatric disorder related to paranoia, personality disorder [27], or somatoform disorder. Some investigators recently proposed that brain dysfunctions might be involved [1, 32, 33].

Most patients with PBS do not have severe psychiatric comorbidities [31]. Watanabe, et al. [31] reported that the frequency of psychiatric comorbidities was significantly lower in PBS occurring with a dental trigger than in PBS without a specific trigger. In addition, patients without a psychiatric comorbidity showed significantly better outcomes than those with a psychiatric comorbidty. After useless dental treatment is stopped [32], antidepressants or aripiprazole may be effective for managing the symptoms of patients with PBS. In addition to clinical studies, brain-imaging studies are expected to clarify the pathophysiology of PBS [34].

### Disturbances in oral sensations

A notable number of patients visit dentists because of unusual oral sensations without evident cause. Patients complain of abnormal sensations such as excessive mucus secretion, slimy sensation in the mouth, or a feeling of a foreign body in the mouth, without corresponding pathological findings in the oral cavity. This disorder is called “oral cenesthopathy” [35].

Because of their firm conviction that their annoying symptoms have a somatic basis, the patients with oral cenesthopathy often visit dental clinics rather than consulting a psychiatrist. To make matters worse, the symptoms in most cases are resistant to drug treatment, which results in dentist shopping [36].

For patients with oral cenesthopathy, strategic compartmentalization among medical specialists and regional medical collaborative platforms may be useful, because this approach can consider complaints about other organs, comorbid psychiatric disorders, and the resulting disabilities of these patients. Progress in brain imaging studies [36–38] and development of a tool for assessing psychosomatic symptoms associated with the mouth [39] may promote an organized collaborative approach to these patients.

### Bad breath

Mouth odor is one of the most common problems in modern dentistry. Some patients complain of oral malodor that is imperceptible to others. These patients are considered to have halitophobia (delusional or psychosomatic halitosis). A related disorder, olfactory reference syndrome (ORS), which is called “jikoshu-kyofu” in Japan, is a condition in which a person mistakenly believes he or she exudes an unpleasant odor [40, 41]. Phillips KA [42] reported that 75 % of his ORS patients were preoccupied with “bad breath”. These patients seem to actually have halitophobia.

Halitophobic patients often visit dental offices to determine if their halitosis originates from dental problems. Many investigations of patients with bad breath have involved the removal of anaerobic microbes or volatile sulfur compounds and have avoided the mental problems of patients with halitophobia. Therefore, most dentists only evaluate bacterial activity in the mouth and repeatedly clean the teeth and tongue. These procedures are not essential for patients with halitophobia and are usually not helpful.

Because halitophobic patients tend to pursue organic causes of their bad breath, providing them with psychological treatment is very difficult. The patient interprets an immediate psychiatric referral as a sign that the dentist believes that the complaint is “psychogenic”: The patient abruptly leaves the dentist and never visits the psychiatrist. Ideally, the dentist can explain the need for treating the psychosomatic aspects of the complaint and can prescribe a selective serotonin reuptake inhibitor along with cognitive therapy [43].

Halitophobia differs from other oral psychosomatic disorders in two major respects. The first is that the main aspect of the patient’s suffering is “taijin-kyofu” (anthrophobia) rather than abnormal oral sensations. Second, most halitophobic patients are adolescents. Future treatment of these patients will require a new clinical approach that is validated for effectiveness.

### Management of dental patients with MUOS

Patients with the MUOS discussed in this review tend to develop another symptom after their previous symptom is ameliorated. Even with obvious improvement, these patients never accept the evidence of improvement and continue to complain of minimal residual symptoms. Patients insist on unique treatment plans, never listen to the advice of specialists, and make repeated requests that the dentist perform as they planned, convinced it is the best solution for their dental problems. Most dentists tire of refusing the patient’s request, which therefore results in more dental treatment than is necessary.

Patients with MUOS may have underlying cognitive distortions that cause them to continue to complain of residual malfunction without regard to obvious improvement as a result of treatment. Although the efficacy of treatment with antidepressants is certainly important, patient-dentist interactions are more critical [44]. Understanding the patient’s mental and oral status plus the use of therapeutic techniques that take into account the interactions between mind and mouth are essential to psychosomatic dentistry.

Psychiatric referral is often difficult and usually not helpful [4]. Such patients generally do not accept such referrals. In addition, psychiatrists do not understand oral complaints without a thorough knowledge on dentistry. To make matters worse, psychiatrists also dislike patients with persistent dental complaints, wanting no business with them.

However, we have reported that approximately 20 to 30 % of patients with MUOS are thought to have actual psychiatric conditions such as depression, bipolar disorder, and severe obsessive-compulsive disorder. Dentists should acquire sufficient training to be able to recognize these mental disorders [45] so that patients can be referred to the appropriate specialist.

Currently, these patients are shunted between dentists and psychiatrists, who shift the responsibility to one another. At least for the time being, it appears that dentists cannot proactively avoid treating patients with MUOS.

## Conclusions

Dentists have been struggling because of the increasing prevalence of MUOS and have been asked to adopt a new treatment approach and to leave behind “brainless dentistry” or “mindless dentistry”. In collaboration with specialists in psychosomatic medicine, the pathophysiology of MUOS should be investigated, with a focus on brain-mouth interactions. The education of dentists who are able to treat not only teeth, but also the patient’s psychosomatic oral discomfort is an important priority.
